# Multi-Scale Finite Element Simulation Framework for Deformation and Damage of Large Structure Under Complex Loadings

**DOI:** 10.3390/ma19132800

**Published:** 2026-07-01

**Authors:** Cheng Li, Chengqi Sun

**Affiliations:** 1 State Key Laboratory of Nonlinear Mechanics, Institute of Mechanics, Chinese Academy of Sciences, Beijing 100190, China; licheng@imech.ac.cn; 2 School of Engineering Science, University of Chinese Academy of Sciences, Beijing 100049, China

**Keywords:** multi-level nested sub-model, finite element analysis, crystal plasticity finite element, large structures, complex loadings

## Abstract

**Highlights:**

- Displacement boundary transferring is an effective method for a multi-level nested sub-model in large structures.
- A multi-level nested sub-model combined with CPFEM could be used for local micro-deformation and damage evolution analysis under complex loadings.
- Integrating the sub-model with CPFEM could accurately predict the fatigue life of metallic materials under complex loadings.

**Abstract:**

This paper establishes a multi-scale nested sub-modeling finite element simulation framework for the deformation and damage analysis of large-scale structures under complex loading conditions. By sequentially transferring displacement solutions from the global model to local sub-models, the framework enables progressive high-resolution analysis from the macroscopic scale (>10 m) down to the microscopic scale (~1 μm), thereby significantly improving solution accuracy in critical regions while maintaining computational efficiency. The proposed approach is validated on a shell structure subjected to hydrostatic pressure and on a plate with a central crack. The results show that the relative errors of stress and strain along specified paths in the shell structure are within 5%, while the relative errors of the stress intensity factor along the crack front in the cracked plate are also below 5%. Furthermore, the framework is integrated with the crystal plasticity finite element method, and a fatigue indicator parameter model based on the accumulated equivalent plastic strain is established to predict the shear fatigue life of Ti-6Al-4V ELI titanium alloy. The predicted fatigue lives are in good agreement with experimental data, with all errors below 10%. This study demonstrates that the proposed sub-modeling method can accurately transfer multi-scale mechanical responses and achieve localized refinement analysis of large-scale structures and can be effectively used for crystal plasticity simulations and fatigue life assessment.

## 1. Introduction

The failure of actual components often originates from difficult-to-detect microscopic damage [1,2,3]. From a macroscopic perspective, high-stress regions or geometric discontinuities in structures generally serve as weak points for damage initiation or failure [4,5]. At the microscopic level, the damage formation is closely related to the microstructure of materials and localized stress concentration [6,7,8]. Therefore, a deep understanding of the multi-scale process of deformation and damage evolution is essential for accurately predicting or evaluating the performance of structures in service [9].

Numerical simulation has become one of the most important ways to gain insight into the microscopic deformation mechanism and damage evolution. With a cross-scale numerical model, the mechanical behavior at different scales can be connected and the influence of microstructure (e.g., grain size, orientation, etc.) on the deformation and damage in structures can be further analyzed. For example, the crystal plasticity finite element method (CPFEM) has been a pivotal approach in studying cross-scale damage evolution, which can characterize local deformation at the microscopic scale and elucidate the underlying damage mechanism [10,11,12,13,14]. Nevertheless, this method generally rests on the assumption that the loading conditions in the microscopic model are equivalent to macroscopic experimental loads. This simplified treatment overlooks the localized stress concentrations arising from geometric discontinuities in practical engineering structures. Consequently, it provides an inaccurate representation of the true local stress state, which hinders the accurate characterization of localized deformation and damage evolution mechanisms.

Kumar et al. [15] investigated the creep-fatigue damage behavior of a titanium alloy using a multi-scale approach. The model parameters were first calibrated with experimental data at the specimen scale and then used on the engine disk component to identify critical damage regions at the microstructure scale. A simplification in this method, however, was the direct application of the disk’s 0.4% peak strain as a uniform boundary condition for the microscopic model. Shi et al. [16] further employed sub-models as simplified crack analysis tools for turbine disc defect assessment, where complex structures are reduced to plate-based crack models with equivalent stress idealizations. Both approaches, however, involve simplifications that may not fully capture the actual local stress states arising from geometric discontinuities in complex structural components. To accurately and efficiently simulate the material behavior at the microscopic scale, some researchers [17,18] divided the macroscopic model into distinct regions. A crystal plasticity model was applied to the local area that was prone to large deformations, whereas a conventional elastic-plastic model was used in the far field where significant plastic deformation was absent. A major limitation in this method was that the differences between the two constitutive models could induce stress discontinuities at interfaces, which led to numerical instability and reduced the reliability of simulation results. Park et al. [19] developed a dual-scale finite element model to investigate the failure behavior of steel with a hole. In their method, the macroscopic deformation was first calculated by using an elastic-plastic constitutive model. Then, the resultant local deformation was applied as boundary conditions for a separate microscopic-scale simulation. This framework provided a way to link the deformation at the macroscopic scale and the microstructural response.

Although existing approaches have played an important role in capturing the influence of microstructural characteristics (such as grain size and orientation) on stress, deformation, and damage evolution, they are generally limited to treat the simplified equivalent loading in the microscopic model and hardly handle complex loadings, particularly in local critical or interested regions of large-scale structures [8]. This study integrates a multi-level nested displacement-transfer sub-modeling strategy with CPFEM to establish a continuous, high-fidelity simulation framework from macroscopic engineering structures down to the grain scale. Compared with existing approaches, the key advantage of this framework lies in its ability to ensure that the microscopic model captures the local stress concentrations present in actual structures, rather than relying on idealized uniform loading assumptions. Meanwhile, sub-models at each level can independently select appropriate constitutive models according to their respective length scales, thereby achieving a balance between computational efficiency and fidelity. First, a multi-level nested sub-modeling strategy is introduced, covering scales from the macroscopic level down to the microscopic level. The proposed framework is then validated on a large shell structure under hydrostatic pressure and on a plate with a central crack by comparison with the global model. Finally, as an application example, the framework is applied to predict the shear fatigue life of the Ti-6Al-4V ELI titanium alloy by integrating experimental data with CPFEM simulations.

## 2. Methodology and Implementation

### 2.1. Methodology

**Figure 1:**
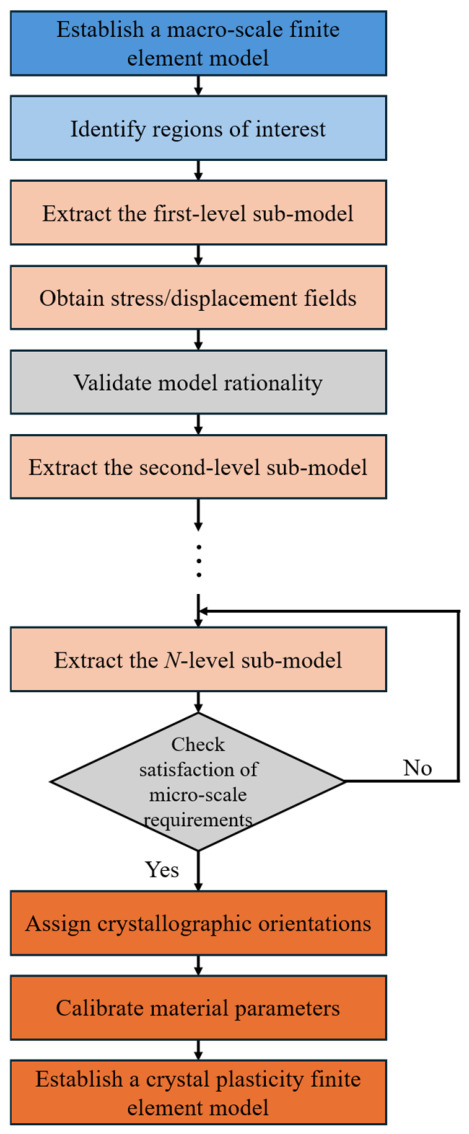
Schematic of the multi-scale finite element simulation framework.

The method in this work could cover a large-scale range, which is from the macroscopic level (e.g., larger than ~10 m) down to the microscopic level (~1 μm), as illustrated in Figure 1. To achieve high accuracy without compromising computational efficiency, a nested multi-level sub-model strategy is adopted, and it proceeds as follows. First, a macroscopic finite element model of the structure is established and solved to obtain the global displacement and stress fields. A region containing the local critical or interested region is then extracted to construct the first-level sub-model, with the global displacement field mapped as boundary conditions onto its cut boundaries. Solving this sub-model provides refined displacement and stress distributions. Subsequently, a smaller region containing the local critical or interested region is extracted from the first-level sub-model to build a second-level sub-model, where the displacement field from the preceding level is prescribed as boundary conditions. This iterative process continues until the Nth-level sub-model containing the local critical or interested region is sufficient for the calculation and characterization. It should be noted that the choice of the material constitutive model depends on the specific application. In the following validations and applications, linear-elastic, elastic-plastic, and crystal plasticity models are adopted as appropriate.

By transferring displacement boundaries across multiple scales, it effectively inherits cross-scale information and enables mesh refinement, thus achieving an optimal balance between computational efficiency and numerical accuracy and making it suitable for the simulation of large-span multi-scale mechanical behavior. This method enables high-resolution solutions in local regions of interest within complex structures and provides flexibility for the sub-model to adopt constitutive models and element types that differ from those used in the global model. In this study, CPFEM is adopted at the microscale, which can characterize the influence of grain size and orientation on the deformation and damage evolution and simulate the microscopic failure mechanism. A schematic of the multi-scale finite element simulation framework is presented in Figure 1.

### 2.2. Multi-Level Nested Finite Element Sub-Model for Large Structure

**Figure 2:**
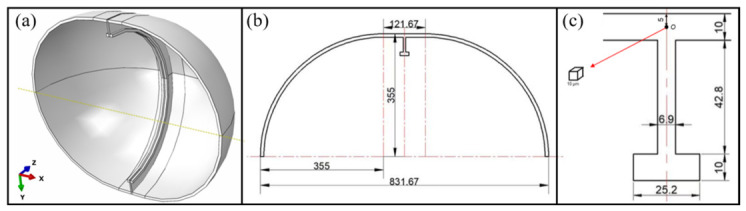
Model of titanium alloy shell structure. (**a**) Three-dimensional solid model; (**b**) two-dimensional schematic (unit: mm); (**c**) magnified view of the local region (unit: mm).

Here, the multi-level nested finite element sub-modeling method is demonstrated by studying the mechanical behavior of an interested region in a meter-scale titanium alloy shell structure under hydrostatic pressure. The model of the structure is shown in Figure 2. It has an internal radius of 355 mm and features an I-shaped stiffener at the mid-section of the shell. In this study, a cube with a side length of 10 μm is considered, which is located in the central region where the stiffener connects to the shell, as shown in Figure 2c. The center of the cube is 5 mm from the outer surface of the shell structure. The shell is assumed to be isotropic, and a linear-elastic constitutive model is employed in the global analysis. The elastic modulus and yield strength are determined from uniaxial tensile tests [20]. Poisson’s ratio ν and density are standard material properties of a titanium alloy. The material parameters are listed in Table 1.

**Figure 3:**
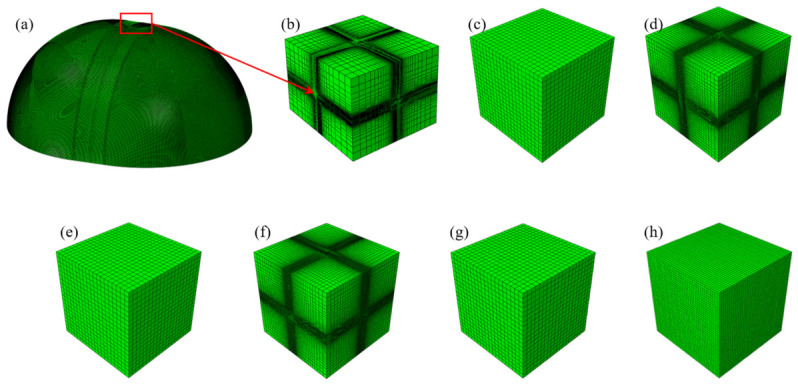
Multi-scale finite element models and mesh generation. (**a**) Global model; (**b**) sub-model-1; (**c**) sub-model-2; (**d**) sub-model-3; (**e**) sub-model-4; (**f**) sub-model-5; (**g**) sub-model-6; (**h**) sub-model-7.

Considering the symmetry in both geometry and loading, a half-symmetry model is adopted to reduce the computational cost. Then, the multi-level sub-model strategy is employed, which comprises four levels ranging from the global macroscopic model to the local microscopic model, as shown in Figure 3. Within these four levels, a total of seven sub-models (sub-model-1 to sub-model-7) are constructed. The multi-level nested sub-modeling procedure is then carried out as follows. First, the global model is solved to obtain the displacement field. A cuboid region of interest is extracted to build the first-level sub-model (sub-model-1), with the global displacement field mapped onto its cut boundaries as boundary conditions. Then, within sub-model-1, a smaller cubic region is extracted to construct the second-level sub-models. At this level, two sub-models (sub-model-2 and sub-model-3) are created with identical geometry but different mesh densities: sub-model-2 retains the coarse mesh from the corresponding region in sub-model-1, while sub-model-3 uses a refined mesh. The displacement field from sub-model-1 is applied as boundary conditions to both sub-models. This nested procedure is repeated to obtain the third-level sub-models (sub-model-4 and sub-model-5) and the fourth-level sub-models (sub-model-6 and sub-model-7). All sub-models are cubic, except sub-model-1, which is a cuboid, and their centers are located 5 mm from the outer surface of the shell structure. Detailed dimensions and mesh information are provided in Table 2. The purpose of having two sub-models at the same level is to compare the effect of mesh refinement on the solution accuracy.

**Figure 4:**
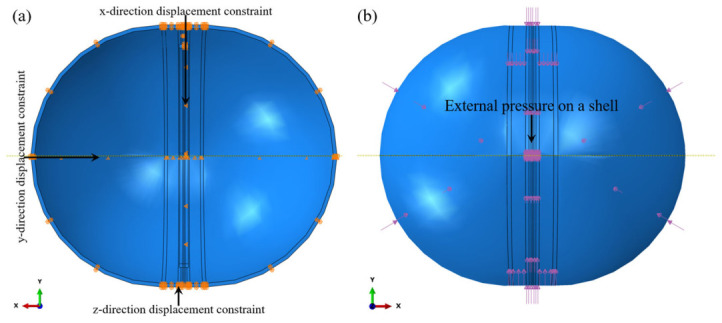
Global model. (**a**) Boundary conditions; (**b**) loading conditions.

To simulate the actual loading conditions, a hydrostatic pressure of 15 MPa is applied to the external surface of the shell structure. The corresponding boundary and loading conditions are illustrated in Figure 4a and Figure 4b, respectively.

### 2.3. Multi-Level Nested Finite Element Sub-Modeling for Cracked Structure

**Figure 5:**
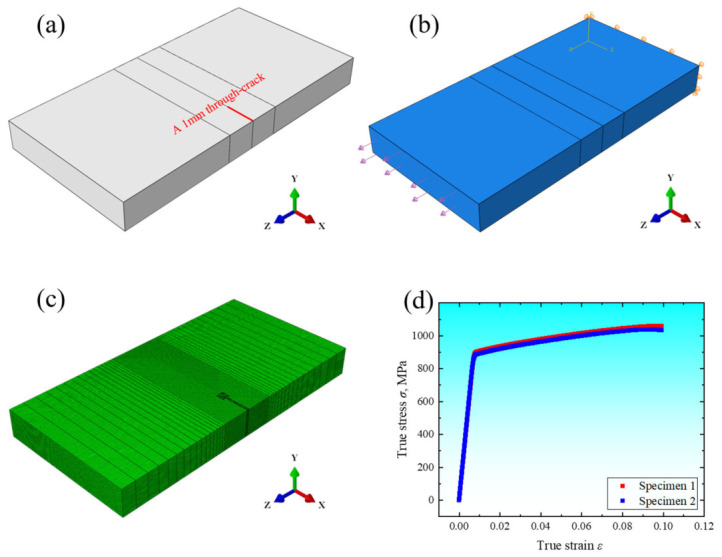
Finite element model of a plate with a crack and stress–strain curves. (**a**) Geometric model; (**b**) boundary and loading conditions; (**c**) mesh of the global model; (**d**) stress–strain curves of Ti-6Al-4V ELI titanium alloy.

Here, the sub-modeling technique is used for the crack analysis. Figure 5a shows the model of a plate with a central through-the-thickness crack. The plate has overall dimensions of 10 mm in length, 5 mm in width, and 1 mm in thickness. A crack with a length of 1 mm is introduced along the center of the plate’s long edge.

A combination of Zencrack and Abaqus is adopted for the crack modeling and simulation. The software versions used in this study are Zencrack 9.5-1 and Abaqus 2024. In this workflow, the uncracked geometry and global mesh are created in Abaqus. Zencrack then inserts the crack, generates the refined mesh around the crack front, and drives the crack propagation simulation by using Abaqus as the finite element solver. Abaqus performs the core finite element calculations (e.g., displacements, stresses, and stress intensity factors), while Zencrack evaluates fracture parameters and updates the crack mesh at each propagation step. The process begins with creating the uncracked geometry and global mesh in Abaqus. Zencrack is then used to insert the crack and generate the refined mesh around the crack region. The boundary conditions and loads for the global model are illustrated in Figure 5b. One end of the model is fixed, and the opposite end is subjected to a tensile stress of 10 MPa. The finite element mesh for the global model with a crack is discretized by using C3D20R and C3D10 elements, as shown in Figure 5c. To balance the computational accuracy and efficiency, a mesh size of 0.05~0.5 mm is adopted for the crack-free region, while a refined size of 0.05 mm is utilized near the crack tip.

In the numerical simulation, Material 1 is steel, for which the popular elastic parameters are adopted; Material 2 is a Ti-6Al-4V ELI titanium alloy, whose elastic modulus, true stress, and true strain data were obtained from tensile tests on cylindrical specimens with a diameter of 5 mm and a gauge length of 25 mm. Two tests were conducted, and the average values were used as material input. The resulting stress–strain curve is shown in Figure 5d. In the simulation, a linear-elastic constitutive model (Material 1) and an elastic-plastic constitutive model (Material 2) were adopted, respectively, with the corresponding material parameters listed in Table 3. It is worth noting that the material parameters in Table 3 correspond to different materials and constitutive models. This difference not only reflects the diversity of material selection in practical engineering but also further demonstrates the good universality of the proposed multi-level sub-modeling method, which is applicable to simulations involving different material behaviors and constitutive models.

**Figure 6:**
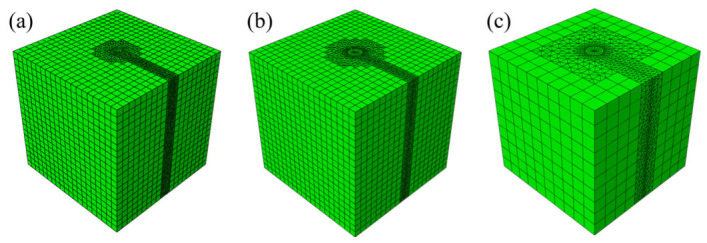
Mesh of sub-models with a crack. (**a**) Sub-model-8; (**b**) sub-model-9; (**c**) sub-model-10.

Three sub-models (sub-model-8, sub-model-9, and sub-model-10) are employed for the progressive analysis. The geometric models and mesh schemes are illustrated in Figure 6. Sub-model-8 is a cubic region with a side length of 0.4 mm, which is extracted from the global model and centered at the crack tip. The crack length is 0.2 mm. Sub-model-9 is a refined cubic region with a side length of 0.04 mm, which is extracted from sub-model-8 and contains a crack length of 0.02 mm. Sub-model-10 is a further refined cubic sub-model (side length: 0.01 mm) with a crack length of 0.005 mm, which is built upon sub-model-9. The mesh parameters are listed in Table 4.

### 2.4. Crystal Plasticity Finite Element Model

Here, the method of crystal plasticity finite element is used for the analysis for microscopic scale. The crystal plasticity constitutive framework is based on that in the literature [21,22,23], which takes into account both slip and deformation twinning for plastic deformation mechanisms. According to the multiplicative decomposition of the deformation gradient, the total deformation gradient **F** is decomposed into the product of an elastic component **F**^e^ and a plastic component **F**^p^, i.e.,

(1) $$F=F^{e}\cdot F^{p}$$

where **F**^e^ is the elastic deformation gradient from lattice distortion and rigid rotation and **F**^p^ is the plastic deformation gradient caused by dislocation slip and deformation twinning.

Taking the time derivative of Equation (1) yields the velocity gradient, **L**, which can also be decomposed into an elastic deformation component, **L^e^**, and a plastic deformation component, **L^p^**:

(2) $$L=L^{e}\cdot L^{p}$$

The plastic velocity gradient, **L^p^**, accounts for three distinct mechanisms: slip in the matrix, twinning in the matrix, and slip in twinned regions [23].

In this study, the Orowan relation is employed to describe the slip shear strain rate [24], i.e.,

(3) $${\dot{\gamma }}_{\mathrm{sl}}^{\alpha }=\rho_{m}^{\alpha }b^{\alpha }v^{\alpha }$$

where $\rho_{m}^{\alpha }$, $b^{\alpha }$, and $v^{\alpha }$ represent the mobile dislocation density, the Burgers vector, and the average dislocation velocity, respectively.

The evolution of twinning shear strain was modeled using a pseudo-slip approach [25], and its rate-dependent flow rule is described as follows:

(4) $${\dot{\gamma }}_{\mathrm{tw}}^{\beta }={\dot{\gamma }}_{\mathrm{tw},0}^{\beta }{\langle \frac{\tau_{\mathrm{tw},\mathrm{eff}}^{\beta }-\tau_{\mathrm{tw},c}^{\beta }}{K}\rangle }^{n}\mathrm{sgn}\left(\tau_{\mathrm{tw},\mathrm{eff}}^{\beta }\right)$$

where ${\dot{\gamma }}_{\mathrm{tw},0}^{\beta }$ is shear strain rate, $\tau_{\mathrm{tw},\mathrm{eff}}^{\beta }$ is the effective shear stress, $\tau_{\mathrm{tw},c}^{\beta }$ is the back stress, and *K* and *n* represent the reference shear stress and strain rate sensitivity parameters, respectively.

The critical resolved shear stress (CRSS) for slip, $\tau_{\mathrm{sl},c}^{\alpha }$, is defined as [26,27,28]:

(5) $$\tau_{\mathrm{sl},c}^{\alpha }=\tau_{\mathrm{sl},0}^{\alpha }+cG_{\mathrm{sl}}^{\alpha }b^{\alpha }{\left(\sum_{\alpha^{\prime }}^{N_{\mathrm{sl}}}\xi_{\alpha \alpha^{\prime }}\rho_{f}^{\alpha }\right)}^{1/2}+K_{\mathrm{HP}}^{\alpha }D_{g}^{-1/2}$$

where $\tau_{\mathrm{sl},0}^{\alpha }$ is the initial CRSS to activate slip, *b^α^* is the length of the Burgers vector, $G_{\mathrm{sl}}^{\alpha }$ is the shear modulus of the slip system, *c* is the material-dependent parameter, $\xi_{\alpha \alpha^{\prime }}$ is the interaction coefficient that describes the interaction between the *α*-th and *α*′-th slip systems, $\rho_{f}^{\alpha }$, $K_{\mathrm{HP}}^{\alpha }$, and $D_{g}$ denote the forest (or sessile) dislocation density on the *α*-th slip system, the Hall–Petch coefficient, and the equivalent grain diameter, respectively.

The CRSS for twinning, $\tau_{\mathrm{tw},c}^{\beta }$, is expressed as [29,30,31]:

(6) $$\tau_{\mathrm{tw},c}^{\beta }=\tau_{\mathrm{tw},0}^{\beta }+G_{\mathrm{tw}}^{\beta }b_{\mathrm{tw}}^{\beta }\sum_{\alpha }^{N_{\mathrm{sl}}}C^{\beta \alpha }b^{\beta }\rho_{f}^{\alpha }$$

where $\tau_{\mathrm{tw},0}^{\beta }$ is the initial CRSS to activate twinning, $G_{\mathrm{tw}}^{\beta }$ is the shear modulus of the twin system, $b_{\mathrm{tw}}^{\beta }$ is the length of the Burgers vector for the twin system, and $C^{\beta \alpha }$ is the latent hardening matrix that describes the interaction between the *β*-th twin system and the *α*-th slip systems.

**Figure 7:**
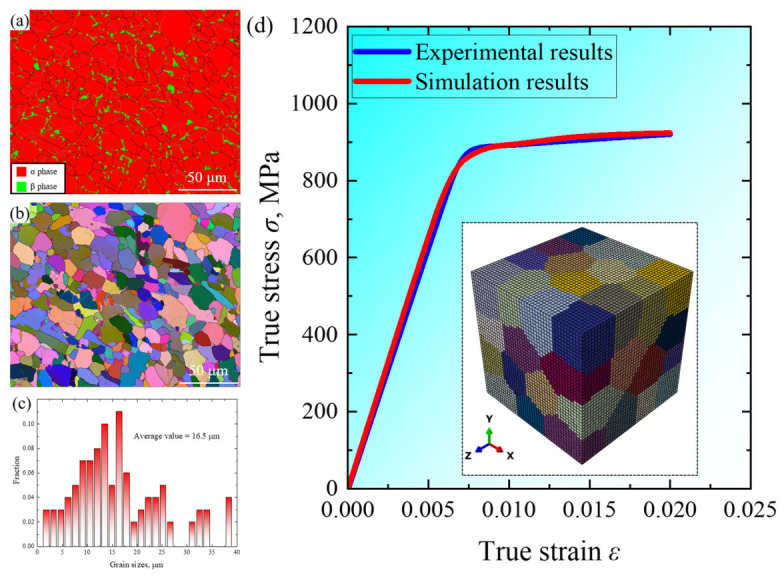
Microstructure and model validation. (**a**) Phase map; (**b**) Euler angle map; (**c**) distribution of grain sizes; (**d**) comparison between monotonic tensile data and simulation results and the schematic of grain morphology for CPFEM.

Electron backscatter diffraction (EBSD) was used to characterize the microstructure of the titanium alloy. The phase map (Figure 7a) indicates α and β phase volume fractions of 95.2% and 4.8%, respectively, while the Euler angle map (Figure 7b) reveals a predominantly equiaxed grain structure. The measured Euler angles were then incorporated into the crystal plasticity finite element simulations to account for polycrystalline anisotropy.

A three-dimensional representative volume element (RVE) was constructed based on the 2D EBSD data shown in Figure 7a,b. The 2D EBSD data were first processed to extract crystallographic orientations, grain size, and phase distribution. A 3D cubic RVE with a side length of 0.05 mm was then constructed using a Voronoi-based grain generation method. The Euler angles obtained from EBSD measurements were directly mapped onto the corresponding grains in the RVE to preserve the crystallographic texture. The phase volume fractions (95.2% α-phase and 4.8% β-phase) were kept consistent with the experimental measurements. Based on the RVE dimensions and the average grain size determined from EBSD (Figure 7c), the RVE was determined to contain 53 grains. The model was discretized using C3D8 elements, resulting in a mesh of 74,088 elements and 79,507 nodes. A mesh independence study was conducted to ensure the reliability of the results. The microstructural characteristics of the RVE (grain size distribution, phase volume fractions, and crystallographic texture) are all derived from EBSD experimental characterization, confirming that the RVE captures the key microstructural features of the Ti-6Al-4V ELI titanium alloy.

To calibrate the material parameters for the CPFEM, the simulated tensile results were compared with experimental data. For the hexagonal close-packed (hcp) α phase, the model incorporated five slip systems and two twinning systems, with its elastic constants defined by five independent parameters: C_11_, C_12_, C_13_, C_33_, and C_44_. For the body-centered cubic (bcc) β phase, the model incorporated three slip systems, with its elastic constants defined by three independent parameters: C_11_, C_12_, and C_44_. These parameters were calibrated using a trial-and-error method based on the experimentally obtained stress–strain curves. Initial estimations were taken from values for similar titanium alloys in the literature [14] and then fine-tuned within reasonable ranges to match the experimental stress–strain curve. The finalized parameters are listed in Table 5, Table 6 and Table 7. As shown in Figure 7d, the simulated stress–strain curve exhibits good agreement with the experimental data, indicating that these parameters accurately characterize the mechanical response of the titanium alloy [32].

## 3. Model Validation and Discussion

### 3.1. Multi-Level Finite Element Sub-Model for Large Structure

**Figure 8:**
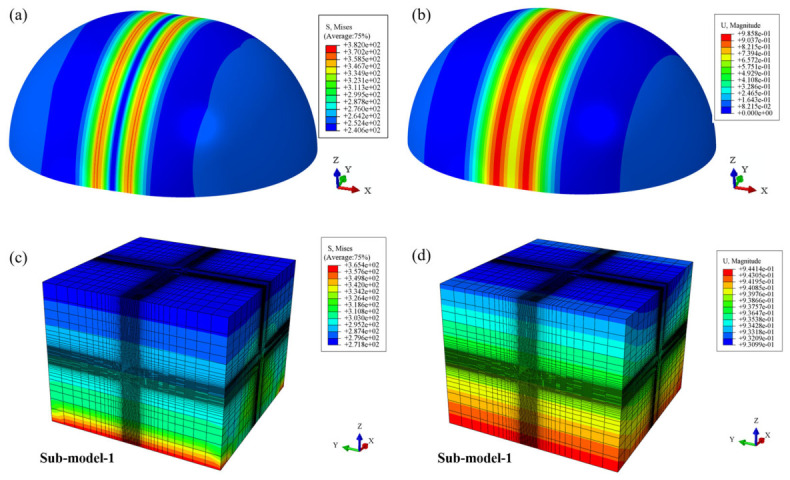
Comparison of simulation results between the global model and the sub-model-1. (**a**) Mises stress distribution of the global model; (**b**) displacement distribution of the global model; (**c**) Mises stress distribution of the sub-model-1; (**d**) displacement distribution of the sub-model-1.

In this section, all models (global model and all sub-models) employ the linear-elastic material model listed in Table 1. Figure 8a and Figure 8b present the Mises stress and displacement distributions of the global model, respectively. The corresponding results from the first-level sub-model (sub-model-1) of the stiffener are shown in Figure 8c,d. The data indicate that the Mises stress in sub-model-1 ranges from 271.8 to 365.4 MPa, with a displacement range of 0.93099 to 0.94414, which agrees well with the results from the corresponding location in the global model.

**Figure 9:**
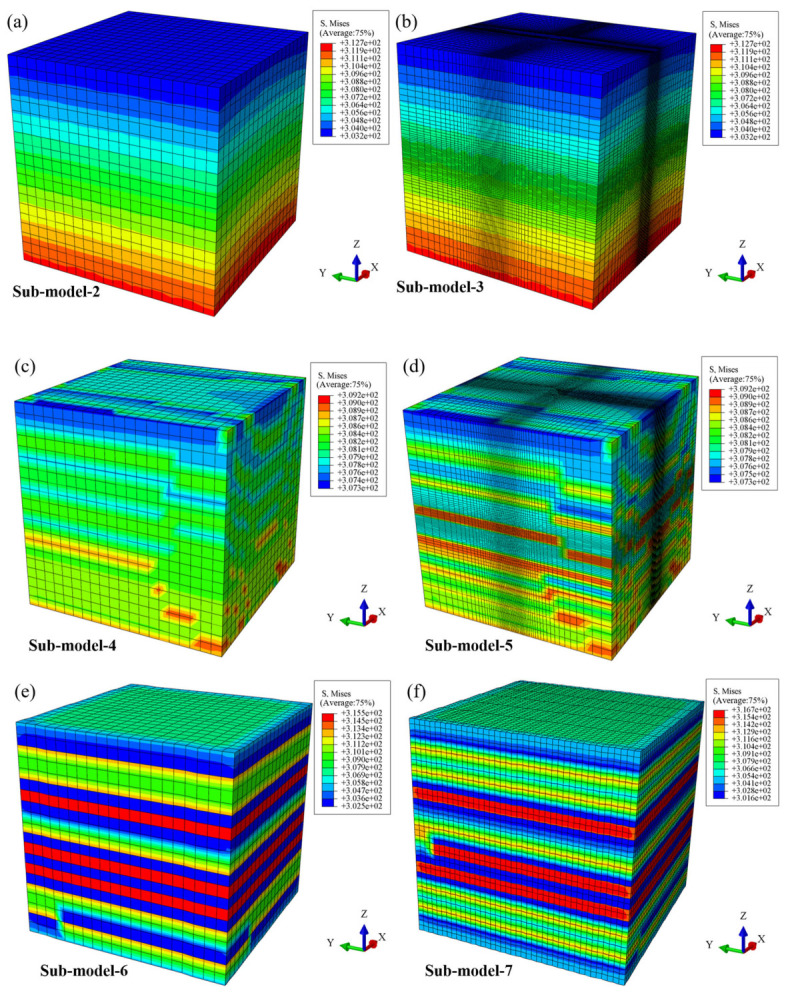
Mises stress distribution in the sub-models. (**a**) Sub-model-2; (**b**) sub-model-3; (**c**) sub-model-4; (**d**) sub-model-5; (**e**) sub-model-6; (**f**) sub-model-7.

Figure 9 illustrates the Mises stress distribution across sub-model-2 to sub-model-7. A comparative analysis of the Mises stress was performed for second-level sub-models under different mesh sizes (i.e., sub-model-2 in Figure 9a and sub-model-3 in Figure 9b). Both sub-models exhibited consistent stress ranges, with maximum and minimum values of 312.7 MPa and 303.2 MPa, respectively. For the third-level sub-models—sub-model-4 (Figure 9c) and sub-model-5 (Figure 9d)—simulations under varying mesh sizes were compared. The results from sub-model-4 and sub-model-5 were nearly identical, showing Mises stress distributions ranging from 307.3 MPa to 309.2 MPa. The comparison of fourth-level sub-models under different mesh sizes—sub-model-6 (Figure 9e) and sub-model-7 (Figure 9f)—revealed maximum Mises stresses of 315.5 MPa and 316.7 MPa, and minimum stresses of 302.5 MPa and 301.6 MPa, respectively.

**Figure 10:**
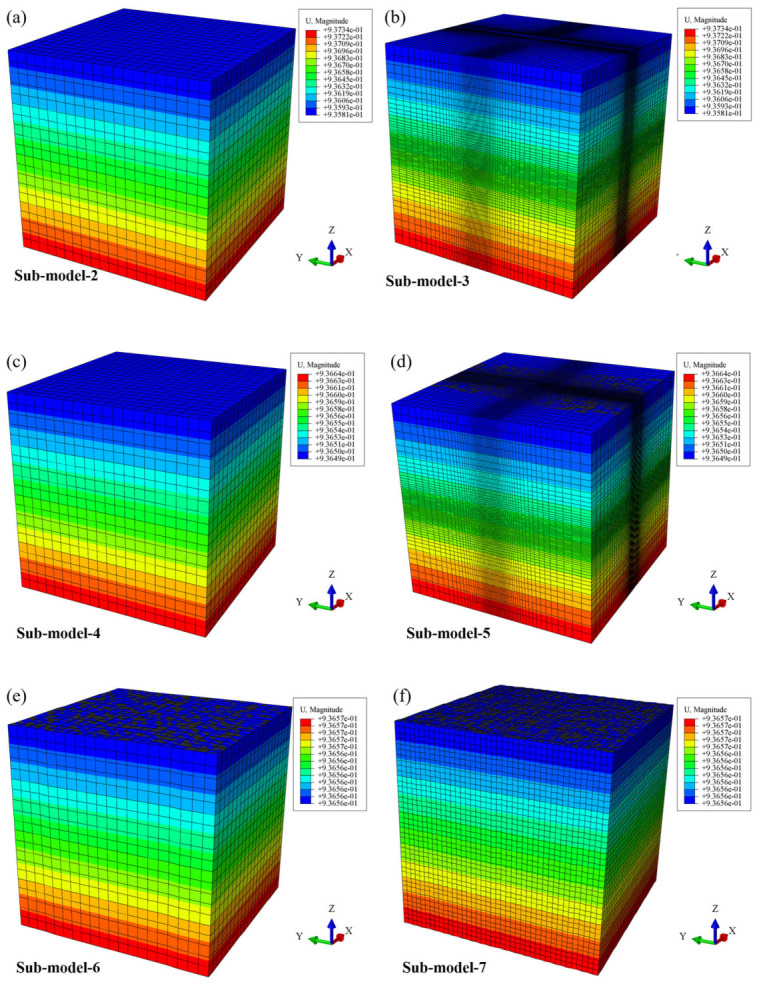
Displacement distribution in the sub-models. (**a**) Sub-model-2; (**b**) sub-model-3; (**c**) sub-model-4; (**d**) sub-model-5; (**e**) sub-model-6; (**f**) sub-model-7.

Figure 10 displays the displacement distributions for the six sub-models. Similar to the Mises stress results, the displacement distributions show minimal variation among sub-models of identical dimensions. The close agreement among these results confirms that mesh density has a negligible influence on the simulated stress and displacement fields for sub-models of the same size.

**Figure 11:**
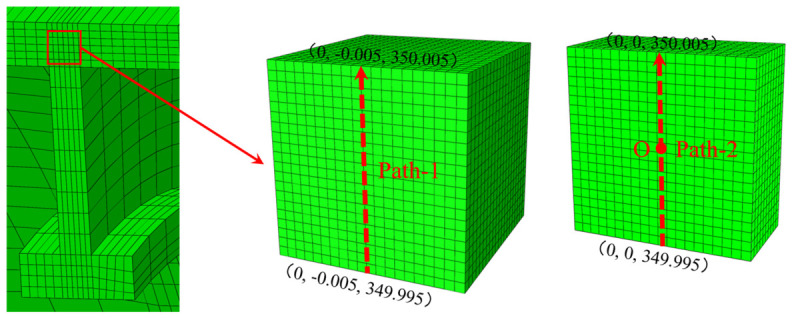
Definition and locations of Path-1 and Path-2 within the model.

Based on the above results, the distributions of extreme Mises stress and displacement values exhibit remarkable consistency across sub-models at the same level. To quantitatively evaluate the discrepancies between the global model and sub-models of various levels, a relative error analysis was performed for the following two paths: a path along the sub-model edge (Path-1) and a path passing through the sub-model center (Path-2). The specific spatial locations of Path-1 and Path-2 are illustrated in Figure 11.

The relative error is calculated as

(7) $$Error=\frac{V_{\mathrm{Sub}\text{-}\mathrm{model}}-V_{\mathrm{Global}\;\mathrm{model}}}{V_{\mathrm{Global}\;\mathrm{model}}}\times 100\%$$

where *V*_Global model_ and *V*_Sub-model_ represent the computational results of the global model and sub-model, respectively.

**Figure 12:**
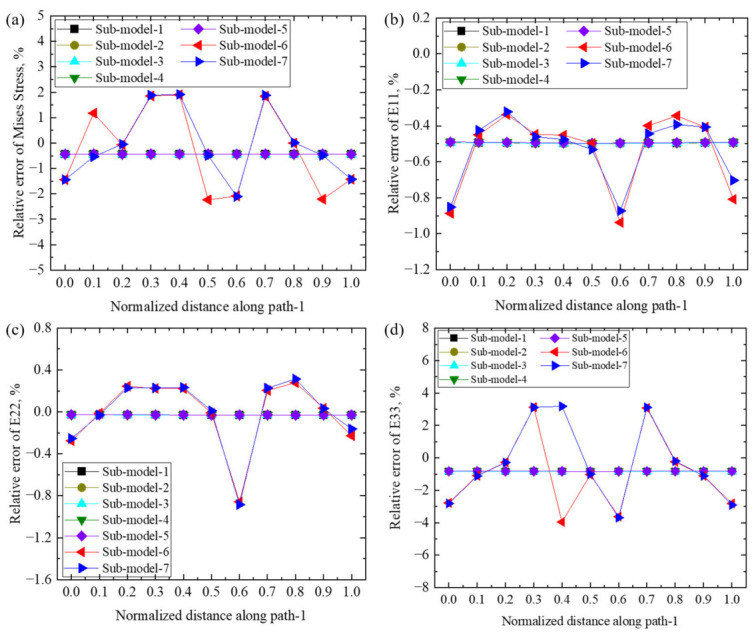
Relative errors of stress/strain along Path-1 of sub-models. (**a**) Mises stress; (**b**) E11; (**c**) E22; (**d**) E33.

**Figure 13:**
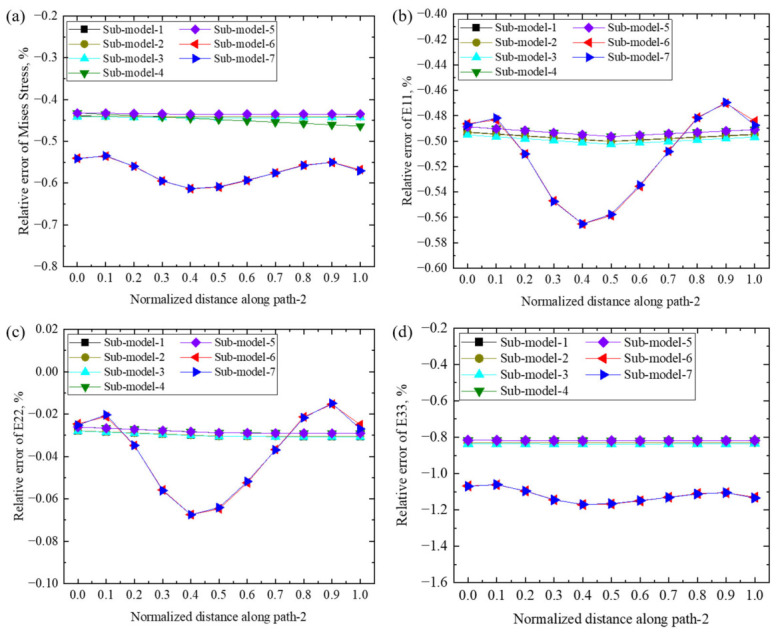
Relative errors of stress/strain along the Path-2 of sub-models. (**a**) Mises stress; (**b**) E11; (**c**) E22; (**d**) E33.

Figure 12 and Figure 13 show the relative error curves of stress/strain along Path-1 and Path-2 in the sub-models, respectively. Notably, only the smallest-scale fourth-level sub-models (sub-model-6 and sub-model-7) exhibit significant fluctuations in relative error, while others remain stable. The relative errors for Mises stress and the normal strains (E11, E22, E33) in X, Y, and Z directions are all below 5% along Path-1, whereas those are all below 2% along Path-2, relative to the global model.

By integrating the comparative analysis of nephograms (Mises stress and displacement) with relative error assessments along critical paths, this study demonstrates that the multi-level sub-modeling technique can effectively obtain reliable stress, strain, and displacement distributions for local areas of a global structure under complex loading. These results confirm the computational accuracy and engineering applicability of the proposed multi-level sub-modeling approach.

### 3.2. Multi-Level Finite Element Sub-Model for Cracked Structure

**Figure 14:**
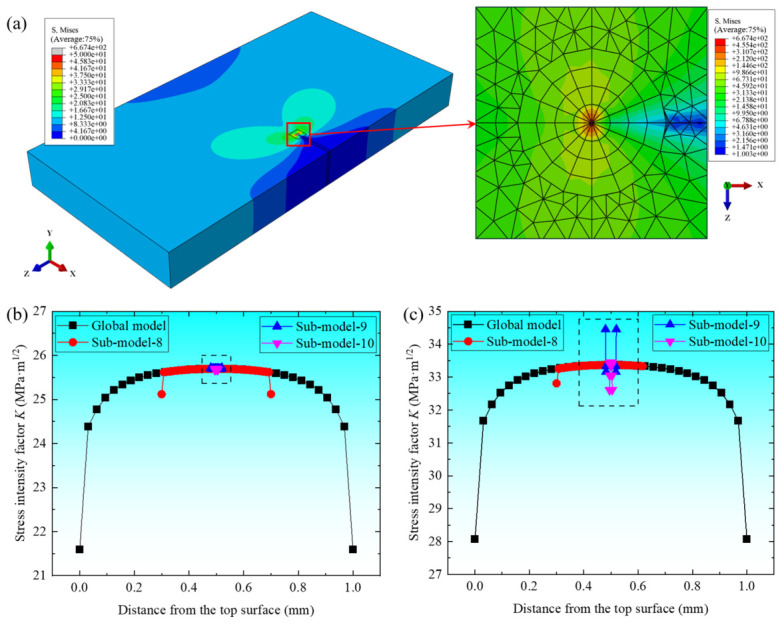
Simulation results of a cracked plate. (**a**) Mises stress distribution; (**b**) comparison of stress intensity factors along the crack front between the global and sub-models using the linear-elastic model; (**c**) comparison of stress intensity factors along the crack front between the global and sub-models using the elastic-plastic model.

In this section, the cracked plate analysis is performed using two material models: a linear-elastic model (Material 1 in Table 3) and an elastic-plastic model (Material 2 in Table 3). The global model and all sub-models consistently use the same material model within each case. Figure 14a shows the nephograms of Mises stress and displacement in the cracked plate. The Mises stress in regions far from the crack is approximately 12.5 MPa. In the crack-tipregion, the stress increases significantly, reaching a maximum value of 667.4 MPa, and exhibits a characteristic butterfly-shaped distribution pattern.

Figure 14b and Figure 14c present the distribution of stress intensity factors along the crack front for the global model and the sub-models under linear-elastic and elastic-plastic constitutive relations, respectively. As shown in the figures, the distributions of the stress intensity factors along the crack front are in excellent agreement between the sub-models and the global model for both constitutive models. The relative error between the two models remains within 5% at every point along the crack front. These results demonstrate that the sub-modeling technique exhibits high computational accuracy and strong applicability for static crack analysis.

### 3.3. Application to Shear Specimen

Validation on typical structures (a shell and a cracked plate) demonstrates that the multi-level sub-modeling technique can transfer mechanical responses from the global model to the local sub-model with high accuracy. To further assess its applicability under complex stress states involving crystallographic features, this study integrates experimental data based on Ref. [32] with CPFEM simulations to predict the shear fatigue life of the Ti-6Al-4V ELI titanium alloy via the sub-modeling approach. The CPFEM calculations were performed using the fatigue life data at nominal stresses of 500 MPa and 400 MPa from Ref. [32], as summarized in Table 8.

**Figure 15:**
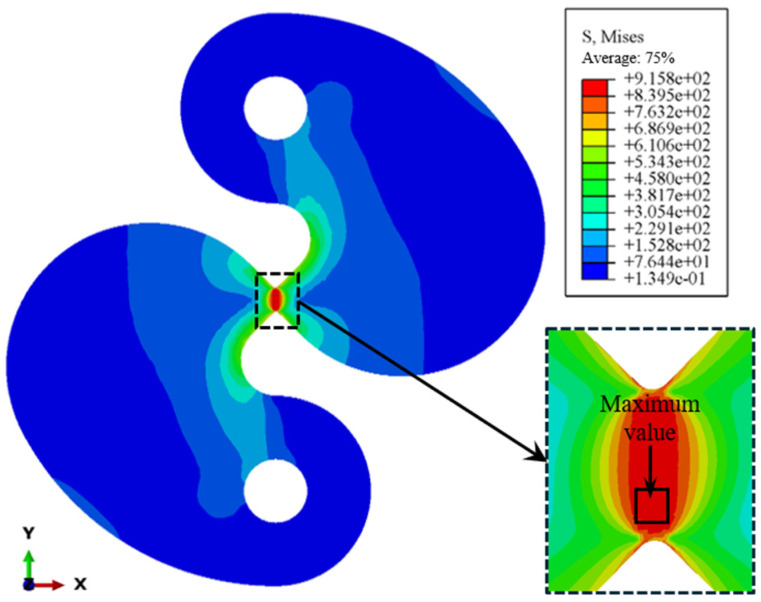
Simulation results of Mises stress for the global model.

In this section, the global model of the shear specimen employs the elastic-plastic material model, while the sub-model employs the calibrated crystal plasticity model. The global model of the shear specimen adopts the same modeling approach in literature [32], where the mesh convergence was verified. The specimen is subjected to shear-dominant fatigue loading with a sinusoidal waveform, a stress ratio of *R* = 0.05, and a frequency of 1 Hz. Figure 15 shows the Mises stress distribution in the shear specimen after 10 loading cycles. The results indicate a pronounced stress concentration in the central region of the specimen, with the maximum Mises stress occurring on its surface. Consequently, this region can be regarded as a potential failure zone. Based on the above stress analysis, a cubic sub-model with a side length of 0.05 mm was extracted from the region of maximum stress. The displacement field from the corresponding location in the global model was applied as the boundary condition for the sub-model, and a crystal plasticity finite element analysis was subsequently performed.

**Figure 16:**
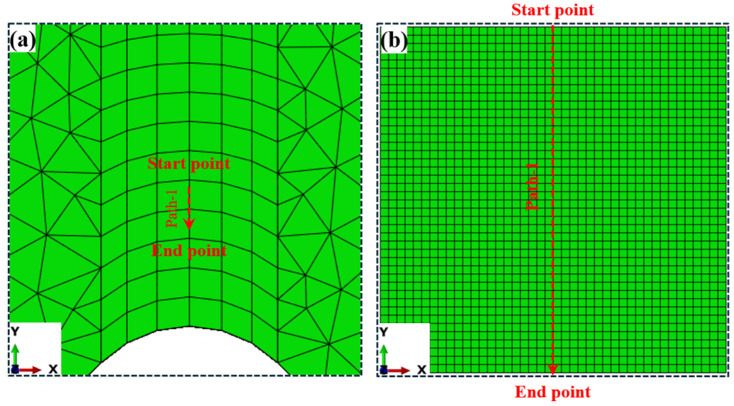
Position of Path-1. (**a**) Global model; (**b**) sub-model.

**Figure 17:**
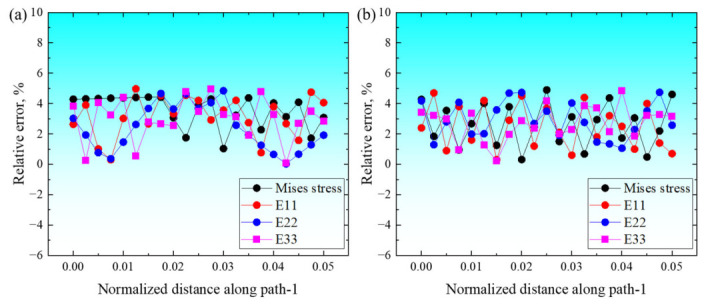
Relative error along Path-1 between the global model and the sub-model. (**a**) Nominal stress of 500 MPa; (**b**) nominal stress of 400 MPa.

To evaluate the accuracy and feasibility of the proposed method, the stress and strain responses obtained from the global model and the sub-model under different stress levels were compared. The corresponding positions of Path-1 in the global model and the sub-model are illustrated in Figure 16a and Figure 16b, respectively. Under nominal stresses of 500 MPa and 400 MPa, the distributions of Mises stress and strain components (E11, E22, E33) along this path are compared in Figure 17a and Figure 17b, respectively. The results show good agreement, with relative errors below 5%, confirming that the sub-modeling approach is applicable to CPFEM calculations.

### 3.4. Fatigue Life Prediction

At the microscopic scale, material anisotropy gives rise to non-uniform strain distributions, making fatigue cracks more likely to initiate in regions of strain concentration or high localization. Therefore, the equivalent plastic strain rate, expressed as follows [33], is adopted in this study to quantify micro-damage accumulation:

(8) $$\dot{p}={\left(\frac{2}{3}L^{P}:L^{P}\right)}^{1/2}$$

The accumulated equivalent plastic strain (*P*_a_), a dimensionless parameter similar to strain, characterizes the progressive accumulation of plastic deformation in polycrystalline materials under cyclic loading. It is computed at each integration point in finite element simulations using the following expression:

(9) $$P_{a}=\int_{0}^{t}\dot{p}\;dt$$

The CPFEM is employed to obtain the *P*_a_ under cyclic loadings, which serves to identify potential fatigue crack initiation sites. Subsequently, a fatigue indicator parameter model is developed to evaluate the fatigue life of the Ti-6Al-4V ELI titanium alloy.

**Figure 18:**
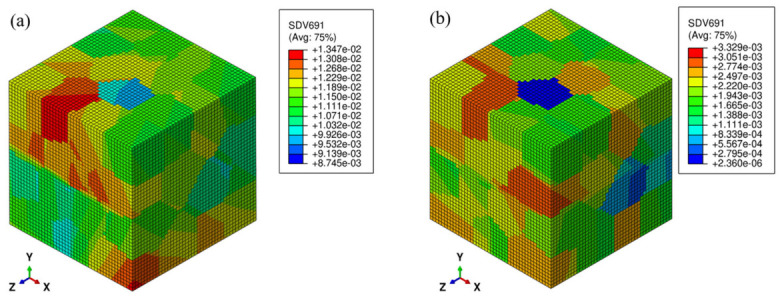
Accumulated equivalent plastic strain from CPFEM simulations under (**a**) Nominal stress of 500 MPa; (**b**) nominal stress of 400 MPa.

Figure 18a,b illustrates the distribution of *P*_a_ in the RVE model after 10 simulation cycles under different loading stresses. The results indicate that *P*_a_ in the RVE increaseswith increasing cyclic loading. Owing to the influence of grain orientation and local stress concentration, *P*_a_ exhibits pronounced spatial inhomogeneity and discontinuity. Tracking the evolution of the maximum *P*_a_ with the number of cycles can be served as a basis for predicting the fatigue life of the material.

To conduct a quantitative analysis, the maximum values of *P*_a_ for each loading cycle are statistically evaluated, and the evolution of the accumulated equivalent plastic strain increment Δ*P*_a_ with loading cycle is obtained. The expression for calculating Δ*P*_a_ is given below:

(10) $$\Delta P_{a}={P_{a}|}_{N}-{P_{a}|}_{N-1}$$

**Figure 19:**
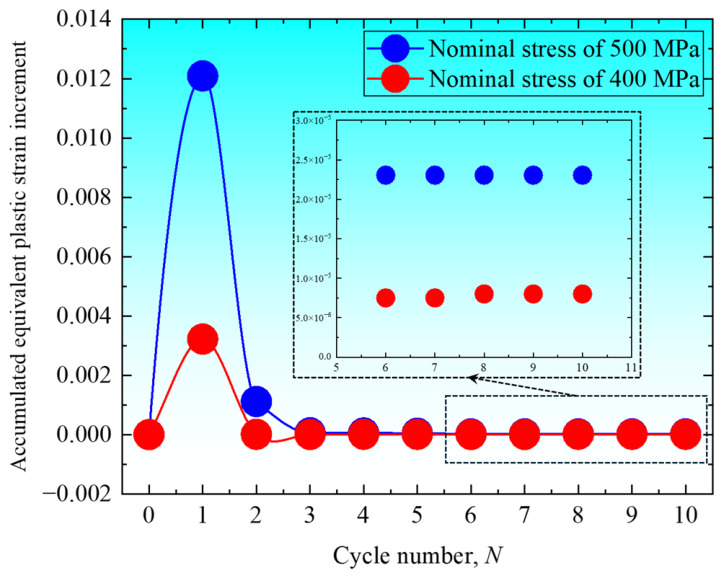
Variation of the accumulated equivalent plastic strain increment with the cycle number.

As shown by Figure 19, Δ*P*_a_ essentially reaches a steady state after about three loading cycles, with the incremental change per cycle stabilizing thereafter. This behavior indicates the stable growth trend of *P*_a_ under cyclic loading.

Previous research [33] has indicated that a critical value of accumulated plastic slip, denoted as *P*_c_, exists at the microscale. When the accumulated plastic slip in a local region of the material reaches this critical value, crack initiation is considered to have occurred. Based on this viewpoint, the crack initiation criterion is expressed as:

(11) $$P_{a}=P_{c}$$

By combining the maximum Δ*P*_a_ from crystal plasticity finite element simulations with experimental fatigue life data at different stress levels, *P*_c_ can be calculated. The preceding analysis shows that *P*_a_ accumulates continuously under cyclic loading, while the per-cycle variation of Δ*P*_a_ can be negligible after three cycles and is treated as constant. Thus, *P*_c_ can be approximately derived as:

(12) $$P_{c}=N_{f}\cdot \Delta P_{a}$$

Once *P*_c_ is determined, it enables the prediction of fatigue crack initiation life under different loading stresses. Crack initiation life is known to account for the majority of the total fatigue life according to relevant studies [34]. Based on the fatigue test data, the *P*_c_ value of the Ti-6Al-4V ELI titanium alloy was calculated to be 0.198 at a reference nominal maximum stress (*σ*_max_) of 500 MPa and fatigue life (*N*_f_) of 8591 cycles. Using this *P*_c_ value along with the Δ*P*_a_ values at different stress levels, the crack initiation life (*N*_p_) under corresponding conditions can be predicted by the following formula:

(13) $$N_{p}=\frac{P_{c}}{\Delta P_{a}}$$

Next, the fatigue life of the Ti-6Al-4V ELI titanium alloy was predicted using the accumulated equivalent plastic strain method. Here, the crack initiation life (*N*_p_) is taken as the fatigue life. For the experimental data at a nominal stress of 400 MPa, two fatigue lives (26,109 and 19,137 cycles) were averaged to 22,623 cycles, and the fatigue life prediction was performed for this average value. Table 9 compares the predicted results with the experimental data. All prediction errors are below 10%, indicating that the sub-modeling technique with the equivalent plastic strain can be used to predict the fatigue life of the Ti-6Al-4V ELI titanium alloy under complex loading conditions.

## 4. Conclusions

This study developed a multi-level nested finite element sub-modeling framework for multi-scale mechanical analysis of large structures under complex loading conditions. The frameworkenables high-resolution simulations in critical regions while maintaining computational efficiency. The main conclusions are summarized as follows:

1. For a large shell structure under hydrostatic pressure, the stress and strain fields calculated by the sub-models along specified paths agree well with those from the global model. The relative errors are within 5%, with most values below 2%, demonstrating that the proposed technique is capable of high-precision local stress and strain computation in small regions.
2. For static crack analysis, the sub-modeling technique exhibits good accuracy and reliability. The stress intensity factors along the crack front obtained from the sub-models show excellent agreement with global model results under both linear-elastic and elastic-plastic constitutive models, with all relative errors below 5%.
3. The proposed framework was further applied to predict the shear fatigue life of the Ti-6Al-4V ELI titanium alloy by integrating experimental data with crystal plasticity finite element simulations. A fatigue indicator parameter model based on accumulated equivalent plastic strain was employed within the crystal plasticity framework. The predicted fatigue lives are in good agreement with experimental data, with all errors below 10%.

## Figures and Tables

**Table 1:** Material parameters [20].

Elastic Modulus (GPa)	Poisson’s Ratio	Yield Strength (MPa)	Density (kg/m^3^)
117.4	0.3	818	4410

**Table 2:** Parameters for global model and sub-models.

Sub-Model Level	Model Name	Model Dimensions	Number of Nodes	Number of Elements
/	Global model	/	1,953,696	1,559,208
First-level	Sub-model-1	10 mm × 10 mm × 7 mm	366,597	351,424
Second-level	Sub-model-2	1 mm × 1 mm × 1 mm	9261	8000
	Sub-model-3	1 mm × 1 mm × 1 mm	300,763	287,496
Third-level	Sub-model-4	100 μm × 100 μm × 100 μm	9261	8000
	Sub-model-5	100 μm × 100 μm × 100 μm	300,763	287,496
Fourth-level	Sub-model-6	10 μm × 10 μm × 10 μm	9261	8000
	Sub-model-7	10 μm × 10 μm × 10 μm	68,921	64,000

**Table 3:** Material properties of Material 1 and Material 2.

Material 1		Material 2			
Elastic Modulus/MPa	Poisson’s Ratio	Elastic Modulus/MPa	Poisson’s Ratio	True Stress/MPa	True Strain
210,000	0.3	124,518	0.3	894.735	0.0
				1040.720	0.075782

**Table 4:** Parameters for sub-models.

Sub-Model Name	Model Dimension	Element Size	Element Type	Number of Elements	Number of Nodes
Sub-model-8	0.4 mm	0.02 mm	C3D20R + C3D10	189,132	332,133
Sub-model-9	0.04 mm	0.002 mm	C3D20R + C3D10	82,448	158,707
Sub-model-10	0.01 mm	0.001 mm	C3D20R + C3D10	39,573	69,965

**Table 5:** Elastic constants in α phase and β phase.

α Phase	*C*_11_/MPa	*C*_12_/MPa	*C*_13_/MPa	*C*_33_/MPa	*C*_44_/MPa
	173,100	98,100	73,600	192,600	49,800
β phase	*C*_11_/MPa	*C*_12_/MPa	*C*_44_/MPa		
	173,100	98,100	73,600		

**Table 6:** Parameters for slip systems in α phase and β phase.

Phase Types	Slip System	$\tau_{sl,0}^{\alpha }$/MPa	*b^α^*/nm	*c*	$\xi_{\alpha \alpha^{\prime }}$ (*α* = *α*′)/(*α* ≠ *α*′)
α phase	{0001}<1$\overset{-}{2}$10>	280	0.295	0.5	0.7/0.1
	{10$\overset{-}{1}$0}<1$\overset{-}{2}$10>	191	0.295	0.5	0.7/0.1
	{10$\overset{-}{1}$1}<1$\overset{-}{2}$10>	363	0.553	0.5	0.7/0.1
	{10$\overset{-}{1}$1}<11$\overset{-}{2}$$\overset{-}{3}$>	241	0.295	0.5	0.7/0.1
	{11$\overset{-}{2}$2}<11$\overset{-}{2}$$\overset{-}{3}$>	295	0.553	0.5	0.7/0.1
β phase	{110}<111>	394	0.295	0.5	0.7/0.1
	{112}<111>	396	0.295	0.5	0.7/0.1
	{123}<111>	398	0.553	0.5	0.7/0.1

**Table 7:** Parameters for twinning systems in α phase.

Twinning System	$\tau_{tw,0}^{\beta }$	*b*^α^/nm	*K*	*n*	${\dot{\gamma }}_{tw,0}^{\beta }$
{10$\overset{-}{1}$2}<$\overset{-}{1}$011>	134	0.0604	350	6	0.1
{10$\overset{-}{1}$1}<10$\overset{-}{1}$$\overset{-}{2}$>	144	0.0877	350	6	0.1

**Table 8:** Stress-life data of Ti-6Al-4V ELI titanium alloy.

Nominal Stress /MPa	Experimental Lives /Cycles
500	8591
500	8133
400	26,109
400	19,137

**Table 9:** Comparison of predicted and experimental fatigue lives.

Nominal Stress/MPa	Error/%
500	5.63
400	9.43

## Data Availability

The raw data supporting the conclusions of this article will be made available by the authors on request.
